# Predicting thyroid involvement in primary Sjögren’s syndrome: development and validation of a predictive nomogram

**DOI:** 10.3389/fimmu.2024.1445916

**Published:** 2024-11-12

**Authors:** Yixuan Yang, Yanyuan Du, Zhaoyang Ren, Qingqing Mei, Mengyao Jiang, Wenjing Liu, Huadong Zhang, Bingnan Cui

**Affiliations:** ^1^ Department of Dermatology, Guang’anmen Hospital, China Academy of Chinese Medical Sciences, Beijing, China; ^2^ Department of Oncology, Guang’anmen Hospital, China Academy of Chinese Medical Sciences, Beijing, China; ^3^ Department of Rheumatology, Guang’anmen Hospital, China Academy of Chinese Medical Sciences, Beijing, China

**Keywords:** thyroid events, nomogram, predictive model, primary Sjögren’s syndrome, risk

## Abstract

**Introduction:**

Patients with Primary Sjögren’s syndrome (pSS) are at a higher risk of thyroid disorders than the general population. This retrospective analysis of 202 patients with pSS was conducted to uncover risk factors associated with thyroid involvement and to create a predictive model for this condition.

**Methods:**

We analyzed 202 patients with pSS from Guang’anmen Hospital, China Academy of Chinese Medical Sciences, with 105 cases of thyroid involvement and 97 without. The Least Absolute Shrinkage and Selection Operator method was used to identify key variables for our risk model. These variables were then subjected to multivariate logistic regression to develop the model. The accuracy of the model was assessed through the C-index, receiver operating characteristic curves, calibration plots, and decision curve analysis, with internal validation via bootstrapping.

**Results:**

High-sensitivity C-reactive protein (HCRP), pulmonary disease, pharyngeal dryness, forgetfulness, night sweats, hyperuricemia, nasal dryness, anxiety, Ro52, and aspartate aminotransferase (AST) were incorporated into the nomogram. The model showed robust discrimination and calibration abilities. Decision curve analysis indicated the clinical utility of our nomogram in intervening on the probability thresholds of thyroid disease.

**Conclusion:**

By integrating HCRP, pulmonary disease, pharyngeal dryness, forgetfulness, night sweats, hyperuricemia, nasal dryness, anxiety, Ro52, and AST, our thyroid risk nomogram can predict the risk of thyroid involvement in patients with pSS, aiding in more informed treatment strategies.

## 1 Introduction

Primary Sjögren’s syndrome (pSS) is a chronic autoimmune disease marked by the infiltration of lymphocytes into the salivary and lacrimal glands. This leads to symptoms such as dry mouth (xerostomia) and dry eye (keratoconjunctivitis sicca), along with the production of autoantibodies (1). The occurrence of pSS varies widely among studies and ethnic groups, from 0.1% to 4.8% (2). While the exact cause of pSS is not fully understood, factors like genetics, hormones, and the environment are believed to be significant in its development. The pathogenesis of pSS involves a complex mix of elements, with environmental triggers possibly initiating the disease in genetically susceptible individuals. Central to pSS are autoimmune reactions and chronic inflammation, which involve the disrupted activation of both innate and adaptive immune responses (3). pSS is not limited to exocrine glands; it can also affect various systemic organs, including the skin, joints, lungs, gastrointestinal tract, kidneys, hematopoietic system, and nervous system (4).

It is important to note that patients with pSS have a significantly higher incidence of thyroid disease compared to the general population, especially for autoimmune thyroiditis (AITD) and subclinical hypothyroidism (5). The occurrence of AITD in patients with pSS is nine times that of the general population (5). Studies have indicated that hypothyroidism is the most common autoimmune disorder in patients with pSS over a 10.5-year follow-up period (6). The association between pSS and thyroid disease suggests shared genetic or environmental factors and similar pathological mechanisms. Thyroid dysfunction in patients with pSS can present as hyperthyroidism, hypothyroidism, or chronic AITD. Moreover, the coexistence of Sjögren’s syndrome (SS) and AITD is relatively common, suggesting a special link between these two diseases in the context of autoimmune disease overlap. For instance, patients with pSS and thyroid disease are often women who test positive for anti-thyroglobulin, anti-follicular cells, and anti-thyroid peroxidase antibodies (7). These thyroid diseases may further influence the clinical presentation and therapeutic decisions for patients with pSS.

Studies have shown varying degrees of thyroid disease prevalence in patients with pSS. Kelly et al. (8) found that 14% of 100 patients with SS had thyroid disorders, with 11% having hypothyroidism and 3% hyperthyroidism. Perez et al. (9) identified thyroid diseases in 45% of 33 patients with SS, including 24% with AITD, 33% with hypothyroidism, and 6% with hyperthyroidism. A study of 160 patients with pSS reported thyroid disease signs in 36%, with 20% diagnosed with AITD and 16% with non-AITD thyroid disorders. Notably, over half of the patients with pSS showed signs of subclinical hypothyroidism (5). Warfvinge et al. (10) examined 63 patients with AITD, with 19 also evaluated for salivary gland morphology and function; 11 had abnormalities in salivary glands, and 31.5% showed clinical and histological features of pSS. Rojas et al. (11), in their analysis of 1083 patients across four autoimmune disease cohorts, found that AITD and SS are the most common co-occurring autoimmune diseases. Several non-controlled studies have reported AITD in SS, with frequencies from 10% to 30% (12). In Lazarus et al.’s study (6), 16% of SS patients developed AITD. The most common clinical manifestation of AITD in SS is hypothyroidism, though subclinical AITD may be even more prevalent. Zeher et al. (13) surveyed 479 patients with pSS for various thyroid diseases, finding thyroid dysfunction in 95 cases (21.25%). The high prevalence of thyroid disease in patients with pSS underscores the importance of screening for thyroid function in this group.

The connection between thyroid disorders and pSS is supported by several theoretical perspectives. Both conditions show similar histological features, such as cellular infiltration dominated by CD4^+^ T cells, forming structures similar to lymphoid follicles without B-cell activation (12). Genetically, they share common HLA class II molecules, including HLA-B8 and HLA-DR3, expressed in thyroid and epithelial cells (12). The combination of HLA-B8 and HLA-DR3 is more prevalent in patients with pSS and AITD, and CTLA-4 gene variations are more common in AITD and other autoimmune diseases like rheumatoid arthritis (14).

Another point strengthening the pathogenic connection between AITD and SS is the pivotal role of chemokines and cytokines in autoimmune inflammatory diseases. Thyroid follicular cells produce CXCL10, which particularly promotes the migration of Th1 cells to the thyroid, triggering the secretion of IFN-γ and TNF-α (15). These cytokines, in turn, stimulate the production of CXCL10, creating a positive feedback loop that intensifies the autoimmune response (16). In Graves’ disease, a subtype of AITD, Th1 cells may play a significant role in the early stages, despite the disease typically being Th2-dominant (17). The expression of CXCL10/IP-10 in thyroid and vascular endothelial cells, along with elevated serum levels of CXCL10, is closely related to the disease activity (18). Similarly, in a mouse model of SS, Th1-associated chemokines are significantly increased in the submandibular gland, whereas Th2-associated chemokines are not detected, suggesting that CXCL10 antagonists might have therapeutic potential by reducing the infiltration of CXCR3^+^ Th1 cells and the production of IFN-γ (19). These findings not only reveal the central role of chemokines in autoimmune inflammatory diseases but also provide molecular evidence for the pathological link between AITD and SS.

Given the multitude of risk factors associated with pSS, predicting the risk of thyroid involvement could prevent thyroid events and improve patient outcomes. Currently, there is no predictive model for thyroid involvement risk in patients with pSS. This study aims to retrospectively identify potential risk factors for thyroid involvement in patients with pSS and to develop a thyroid risk nomogram. The goal is to provide clinicians with a new tool to more accurately identify patients at higher risk of thyroid involvement, allowing for earlier interventions and potentially better patient outcomes.

## 2 Methods

### 2.1 Participants

From January 2022 to December 2022, patients with pSS were diagnosed and followed up in the Department of Rheumatology at Guang’anmen Hospital, China Academy of Chinese Medical Sciences. Inclusion Criteria: All patients met the ACR/EULAR 2016 classification criteria, which are as follows. Patients must have at least one of the symptoms of dry eyes or dry mouth, and must not meet any exclusion criteria. A diagnosis of pSS can be made if the total score of the following five items is ≥4: ①Focal lymphocytic sialadenitis in labial salivary gland biopsy with a focus score of ≥1 focus/4 mm², 3 points. ②Positive anti-SSA/Ro antibodies, 1 point. ③Ocular staining score (OSS) of ≥5 in at least one eye or a van Bijsterveld score of ≥4, 1 point. ④Schirmer’s test result of ≤5 mm/5 min in at least one eye, 1 point. ⑤Unstimulated whole saliva flow rate of ≤0.1 ml/min as measured by the Navazesh and Kumar method, 1 point. (American College of Rheumatology/European League Against Rheumatism classification criteria for pSS: a consensus and data-driven methodology involving three international patient cohorts). Exclusion Criteria: Patients were excluded if they lacked essential data, such as thyroid involvement.

This study was approved by the Ethics Committee of Guang’anmen Hospital, China Academy of Chinese Medical Sciences (Approval No.2022-132-KY) and adhered to the principles of the Declaration of Helsinki. Informed consent was obtained verbally from all patients at the time of their clinical visits. After confirming the research protocol, we conducted a brief telephone follow-up with the participants. Initially, we verified the participants’ identities and provided a concise explanation of the clinical trial, including its design and the approval from the ethics committee. We then obtained verbal informed consent. Upon receiving informed consent, we collected the participants’ information and proceeded with the relevant research. Participants who declined to participate were excluded from the study, and no information was collected from them.

### 2.2 Characteristics

As a retrospective observational study, the sociodemographic and clinical characteristics of our patients were obtained through interviews and physical examinations. Information collected from medical histories included age at initial diagnosis, gender, symptoms, involved systems, and other comorbidities at the first visit. The data were collected by trained clinicians and medical staff to ensure accuracy. Symptoms primarily included dry nose, dry throat, anxiety and irritability, night sweats, forgetfulness and so on. In our study, dry nose is defined as dryness of the nasal mucosa with reduced secretion. Dry throat is defined as reduced salivary secretion and dryness of the pharyngeal mucosa.Anxiety and irritability are defined as encompassing feelings of fear, worry, and restlessness, possibly escalating to panic and a sense of impending doom, as well as an intolerance for waiting, a propensity for excitability, and an exaggerated reaction to setbacks and delays. Night sweats is defined as excessive sweating after falling asleep, which stops upon waking. Forgetfulness is defined as an inability to remember new events or recall one or more past memories. Pulmonary diseases are defined as various conditions affecting lung function, impacting respiratory function, including but not limited to chronic obstructive pulmonary disease, asthma, and interstitial lung disease. Hyperuricemia is defined as serum uric acid levels above 420 µmol/L on a regular diet. The laboratory data for these patients were obtained from standardized tests conducted at the clinical laboratory of Guang’anmen Hospital, China Academy of Chinese Medical Sciences, all of which have been validated. The laboratory information included complete blood count, blood biochemistry, liver and kidney function tests, antibody profiles, and urinalysis. During the follow-up period, thyroid events such as hyperthyroidism, hypothyroidism, thyroiditis, and goiter were recorded. The laboratory tests were conducted under controlled conditions to ensure consistency and reliability. In this study, the complete blood count (CBC) was performed using the Sysmex XN-3000 analyzer (Sysmex Corporation, Kobe, Japan), which combines flow cytometry and impedance technology for accurate measurement of red blood cells, white blood cells, and platelets. Flow cytometry was used for fluorescence labeling and classification of white blood cells, while impedance technology measured cell volume. Blood samples were collected in EDTA tubes and analyzed within one hour of collection to ensure stability and accuracy. Blood biochemistry analysis was conducted using the Beckman AU5800 automated biochemical analyzer (Beckman Coulter, Brea, CA, USA). The AU5800 uses spectrophotometric methods to quantify biochemical parameters, including liver and kidney function, glucose, lipids, and electrolytes. Blood samples were centrifuged at 3,000 rpm for 10 minutes to separate serum for analysis. Antibody profiling was performed using the Freedom EVOlyzer automated ELISA analyzer (Tecan Group Ltd., Männedorf, Switzerland) with validated commercial ELISA kits. Venous blood samples were collected, centrifuged, and analyzed with automated pipetting, incubation, washing, and optical detection at 450 nm. Urinalysis was carried out using the Sysmex UN-2000 system, consisting of the UF-3000 flow cytometry analyzer and the UC-3500 chemical module (Sysmex Corporation, Kobe, Japan). The UF-3000 used fluorescence flow cytometry to analyze urine particles, while the UC-3500 used dry chemistry for biochemical parameters such as glucose, protein, and pH. Urine samples were collected in sterile containers and analyzed within two hours to ensure integrity.

### 2.3 Classification of patients with pSS

Our previous analysis of patients with pSS revealed that the incidence of structural or functional thyroid abnormalities is higher in patients with pSS compared to the healthy population. Consequently, we defined hyperthyroidism, hypothyroidism, thyroiditis, and goiter as thyroid events. Based on the occurrence of subsequent thyroid events during the follow-up period, we categorized the patients into two groups: the thyroid disease group (TD) and the non-thyroid disease group (NTD). Hyperthyroidism is an endocrine disorder characterized by excessive secretion of thyroid hormones, including triiodothyronine (T3) and thyroxine (T4), from the thyroid gland. This condition is typically accompanied by a decrease in serum thyroid-stimulating hormone (TSH) levels. Clinically, hyperthyroidism presents with symptoms such as palpitations, weight loss, excessive sweating, irritability, and other systemic manifestations associated with increased thyroid activity. TSH levels are usually significantly below the normal range, often less than 0.1 mIU/L. Elevated serum free T4 (FT4) and free T3 (FT3) levels indicate excessive secretion of thyroid hormones, confirming the diagnosis of hyperthyroidism (20). Hypothyroidism is a condition characterized by a decreased secretion of thyroid hormones or a reduction in their physiological action, leading to a slowdown in metabolic processes. The disease can be classified into overt hypothyroidism and subclinical hypothyroidism. Overt hypothyroidism is marked by significantly elevated levels of TSH, typically greater than 10 mIU/L, accompanied by decreased levels of FT4. Subclinical hypothyroidism, on the other hand, presents with mildly elevated TSH levels, usually ranging between 4.5 and 10 mIU/L, while FT4 levels remain within the normal range (21). Thyroiditis refers to a group of disorders involving inflammation of the thyroid gland, commonly associated with thyroid dysfunction. Depending on the underlying pathophysiological mechanisms, thyroiditis can be classified into various types, including autoimmune thyroiditis (e.g., Hashimoto’s thyroiditis), subacute thyroiditis, and painless thyroiditis. Hashimoto’s thyroiditis is the most prevalent form of autoimmune thyroiditis, typically presenting with hypothyroidism. Clinically, it is characterized by diffuse thyroid enlargement (goiter). Laboratory findings include elevated serum TSH levels and decreased FT4 levels. Autoimmune markers, such as thyroid peroxidase antibodies (TPOAb) or thyroglobulin antibodies (TgAb), are usually positive. On thyroid ultrasound, the thyroid gland often exhibits an irregular structure and reduced echogenicity, indicative of chronic inflammation (22). Subacute thyroiditis, often caused by viral infections, presents clinically with thyroid pain and tenderness, frequently accompanied by systemic symptoms such as fever, palpitations, weight loss, and sweating, which are indicative of transient hyperthyroidism. Laboratory tests show decreased TSH levels and elevated levels of FT4 and FT3. The erythrocyte sedimentation rate (ESR) is typically elevated. A thyroid radionuclide scan usually demonstrates reduced radioactive iodine uptake, reflective of diminished thyroid function (23). Painless thyroiditis presents similarly to subacute thyroiditis but without pain. It may initially cause transient hyperthyroidism, followed by hypothyroidism. Thyroid function tests reveal a brief period of hyperthyroidism, similar to subacute thyroiditis. Autoimmune markers (TPOAb or TgAb) may be positive, and a thyroid radionuclide scan typically shows decreased radioactive iodine uptake (24).

### 2.4 Data management

All collected data, including laboratory results and clinical observations, were securely stored in an electronic database. Data entry was double-checked to avoid transcription errors. This data management process ensures both the reliability and traceability of the collected information. Only authorized personnel had access to the database, ensuring the protection of patient confidentiality.

### 2.5 Statistical analysis

The sociodemographic and clinical characteristics of our patients are presented as mean (SD), median (interquartile range), or count (percentage) and were analyzed using R software (version 4.3.3; https://www.R-project.org). Comparisons between the two groups were performed using the t-test, Mann-Whitney U test, or chi-squared test as appropriate. The Mann-Whitney U test was used for continuous variables, while Fisher’s exact test was used for categorical variables. Odds ratios (OR) with 95% confidence intervals (CI) and p-values (two-tailed) were used to evaluate these characteristics. We used the Least Absolute Shrinkage and Selection Operator (LASSO) regression model to select the most predictive non-zero features for thyroid events in patients with pSS. Subsequently, we performed multivariate logistic regression on these features and developed a predictive model. This model included all sociodemographic and clinical characteristics with p-values less than 0.05. Additionally, other potential predictive factors were incorporated into the thyroid risk prediction model. Receiver operating characteristic (ROC) curves and calibration curves were used to evaluate the thyroid risk nomogram. Harrell’s C-index was measured to quantify the discrimination ability of the thyroid risk model. Bootstrapping validation (1000 bootstrap resamples) was used to obtain a relatively calibrated model C-index. Calibration curve analysis was performed to assess the clinical applicability of the thyroid risk model by quantifying the net benefit (the proportion of true positive patients minus the proportion of false positive patients, balancing the relative harm of not intervening with the negative impact of unnecessary intervention) across different threshold probabilities in the pSS cohort.

## 3 Results

### 3.1 Characteristics of patients

This study involved the diagnosis and follow-up of 202 patients with primary pSS at Guang’anmen Hospital, China Academy of Chinese Medical Sciences, conducted from January 2022 to December 2022. The study included 8 male and 194 female patients, aged between 21 and 92 years. Based on the occurrence of thyroid events after diagnosis, 97 patients were categorized into TD group, and 105 patients were categorized into the non-thyroid disease NTD group. The characteristics of all study patients (TD and NTD groups) are shown in (**Table 1**). Compared to the TD group, the NTD group had a higher prevalence of pulmonary diseases (87.62% vs 73.20%, *p*=0.009), and significantly higher levels of high-sensitivity C-reactive protein (HCRP) (89.52% vs 59.79%, *p*<0.001). Additionally, the TD group was more prone to hyperuricemia than the NTD group (98.97% vs 93.33%, *p*=0.04). Furthermore, the NTD group exhibited a higher prevalence of dry throat (45.36% vs 31.43%, *p*=0.042), anxiety and irritability (25.77% vs 11.43%, *p*=0.008), forgetfulness (10.31% vs 21.90%, *p*=0.026), and elevated aspartate aminotransferase (AST) levels (88.66% vs 96.19%, *p*=0.041). There were no significant differences between the two groups in other characteristics.

**Table 1:** Clinical characteristics of patients with primary Sjögren’s syndrome.

Characteristics	TD (n=105)	NTD (n=97)	χ²	p
Pulmonary disease,n (%)	92 (87.619)	71 (73.196)	6.733	0.009
	13 (12.381)	26 (26.804)		
Hyperuricemia,n (%)	98 (93.333)	96 (98.969)	4.21	0.04
	7 (6.667)	1 (1.031)		
Dry nose,n (%)	78 (74.286)	81 (83.505)	2.558	0.11
	27 (25.714)	16 (16.495)		
Dry throat,n (%)	33 (31.429)	44 (45.361)	4.149	0.042
	72 (68.571)	53 (54.639)		
Anxiety and irritability,n (%)	12 (11.429)	25 (25.773)	6.934	0.008
	93 (88.571)	72 (74.227)		
Night sweats,n (%)	46 (43.810)	54 (55.670)	2.837	0.092
	59 (56.190)	43 (44.330)		
Forgetfulness,n (%)	23 (21.905)	10 (10.309)	4.96	0.026
	82 (78.095)	87 (89.691)		
AST,n (%)	101 (96.190)	86 (88.660)	4.16	0.041
	4 (3.810)	11 (11.340)		
Ro52,n (%)	16 (15.238)	10 (10.309)	1.092	0.296
	89 (84.762)	87 (89.691)		
HCRP,n (%)	94 (89.524)	58 (59.794)	23.927	<0.001
	11 (10.476)	39 (40.206)		

TD, Thyroid Disease; NTD, non-thyroid disease; AST, Aspartate Aminotransferase; HCRP, High-sensitivity C-Reactive Protein.

### 3.2 Feature selection

**Figure 1:**
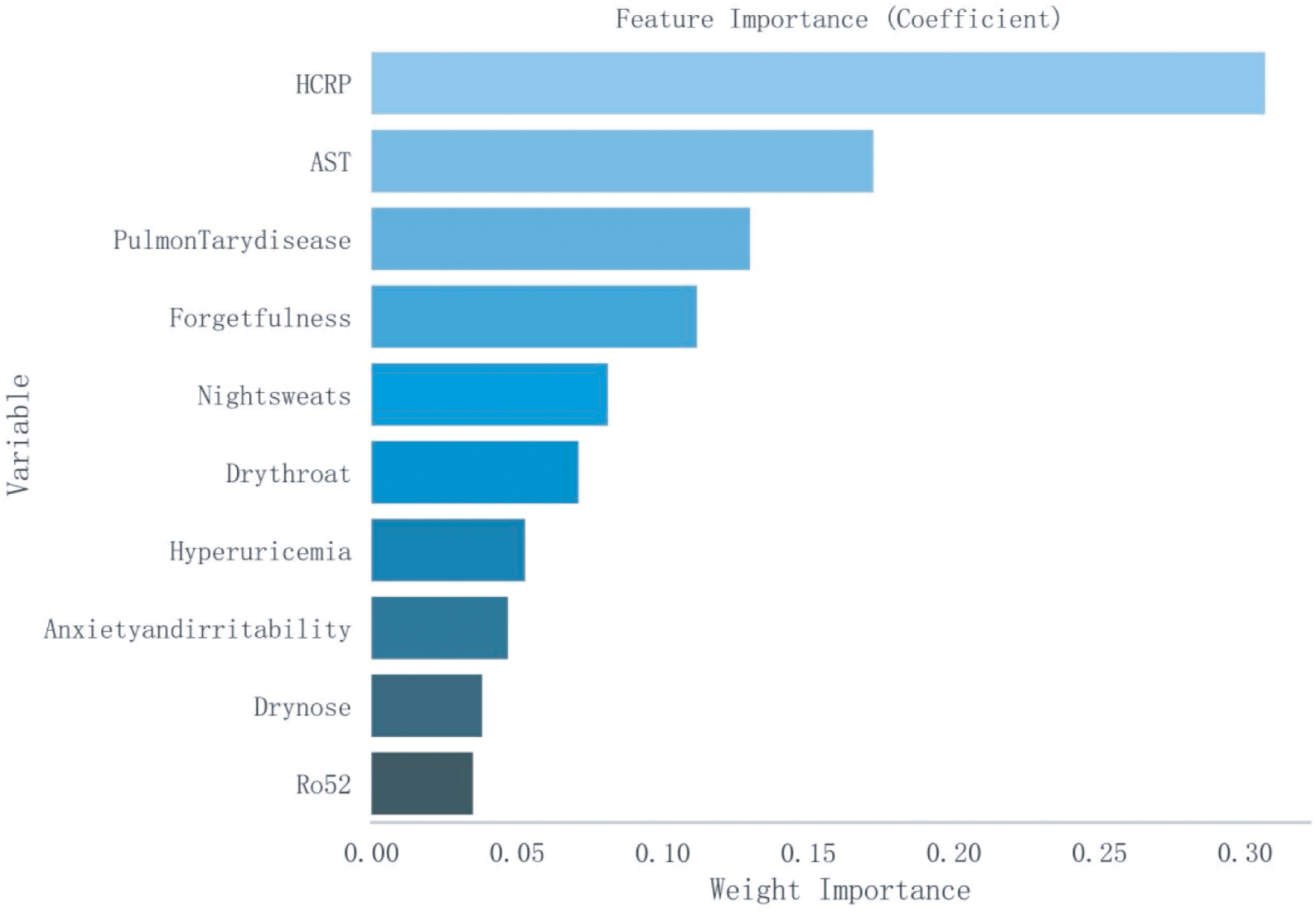
Ranking of Importance of Ten Features. AST, Aspartate Aminotransferase; HCRP, High-sensitivity C-Reactive Protein.

**Figure 2:**
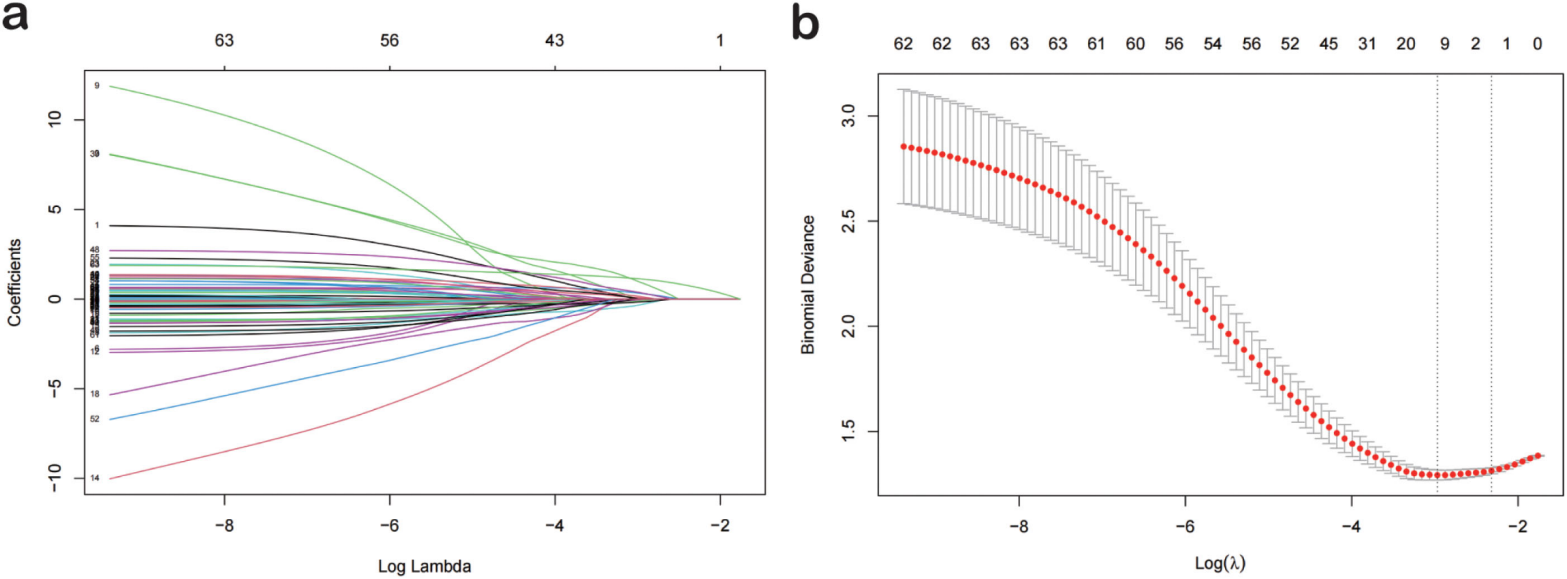
Lasso Logistic Regression for Feature Selection. **(A)** The optimal parameter (lambda) in the lasso model was selected using the minimum criterion via five-fold cross-validation. The partial likelihood deviance (binomial deviance) curve was plotted against the logarithm of lambda. Dashed lines were drawn at the optimal values using the minimum criterion and the 1-SE criterion. **(B)** Lasso coefficient profiles for 77 features. A coefficient profile plot was generated against the logarithm of lambda sequence. A vertical line was drawn at the value selected by five-fold cross-validation, where the optimal analysis resulted in five features with non-zero coefficients. Lasso, Least Absolute Shrinkage and Selection Operator; SE, Standard Error.

Based on a cohort of 202 patients, we employed a LASSO regression model with the following parameters: cross-validation folds (cv) set to 5, maximum iterations (max_iter) set to 1000, tolerance (tol) set to 0.0001, and L1 regularization coefficient (alpha) set to 0.01. Upon fitting the model, the regression coefficients for each feature were extracted. The absolute value of these coefficients indicates the contribution of each feature to the model’s prediction, with larger absolute values signifying greater importance. The importance of features was then ranked by sorting the absolute values of all coefficients (**Figure 1**). This process reduced the 62 sociodemographic and clinical features to 10 potential predictive factors with non-zero coefficients, representing a reduction ratio of approximately 6:1 (**Figure 2**). These features include HCRP, pulmonary disease, hyperuricemia, Ro52, AST, dry nose, dry throat, anxiety and irritability, night sweats, and forgetfulness. HCRP serves as an inflammatory marker that typically escalates in the presence of acute inflammation or infection. Ro-52 antibodies, commonly referred to as anti-Ro-52 antibodies, are a nonspecific indicator often observed in autoimmune diseases. AST is a biomarker for liver function that tends to increase in the blood when liver damage is present.

### 3.3 Identification of significant risk factors for TD

**Figure 3:**
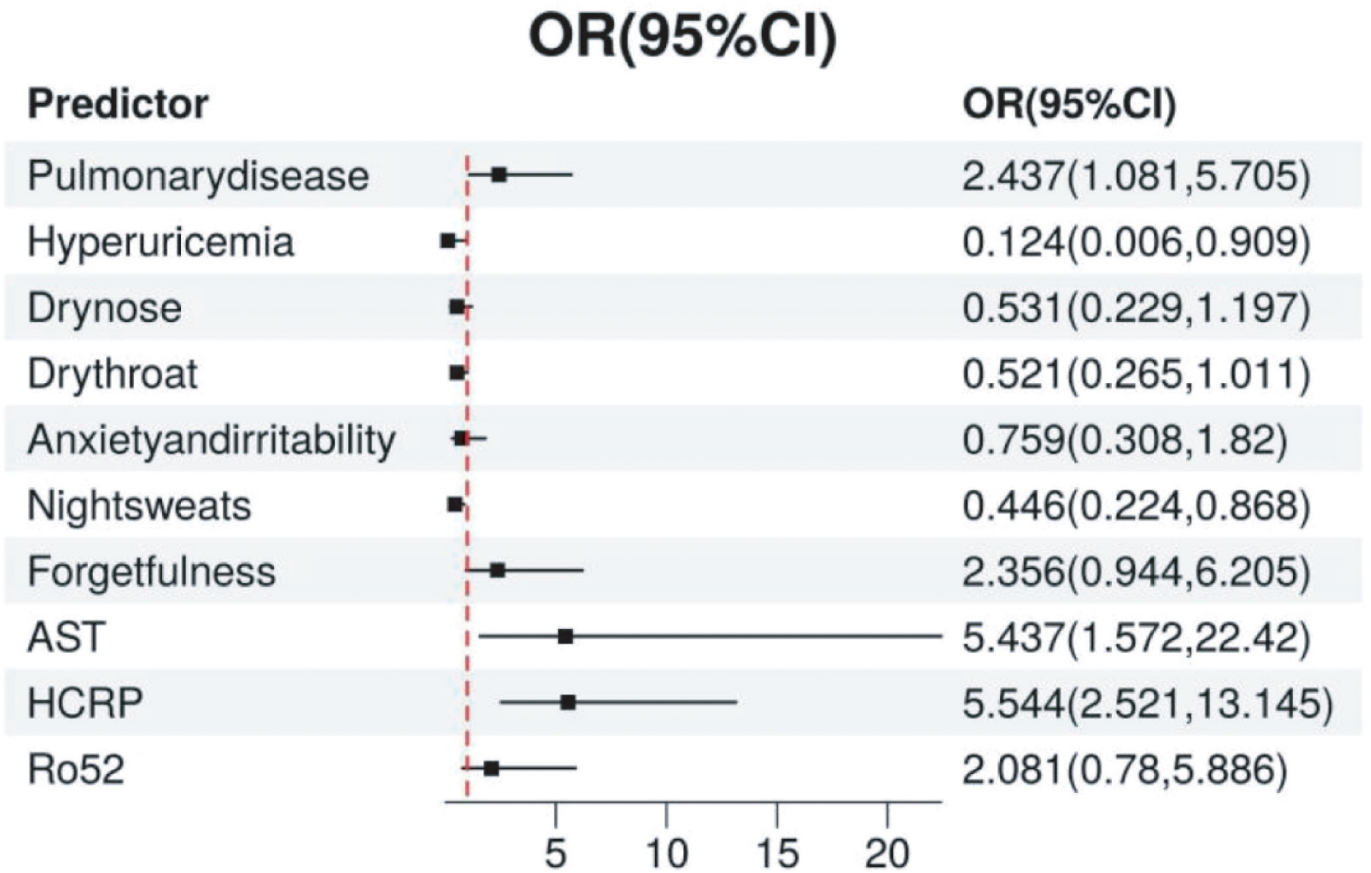
The Relationship Between Various Predictive Variables and TD. The point estimates (odds ratios, OR) and their 95% confidence intervals (CI) for each variable are displayed in the figure. Each point represents the odds ratio for the respective variable, and the horizontal line denotes the 95% confidence interval. TD, Thyroid Disease.

We established a logistic regression model incorporating HCRP, pulmonary disease, hyperuricemia, Ro52, AST, dry nose, dry throat, anxiety and irritability, night sweats, and forgetfulness (**Table 2**). The odds ratios (OR) and 95% confidence intervals (CI) for each factor were expressed using a forest plot (**Figure 3**). Patients with pulmonary disease were more likely to develop TD compared to those without pulmonary disease (OR: 2.437, 95% CI: 1.081–5.705, *p*=0.035). The relationships between hyperuricemia (OR: 0.124, 95% CI: 0.006–0.909, *p*=0.078), dry nose (OR: 0.531, 95% CI: 0.229–1.197, *p*=0.131), dry throat (OR: 0.521, 95% CI: 0.265–1.011, *p*=0.055), anxiety and irritability (OR: 0.759, 95% CI: 0.308–1.82, *p*=0.539), and TD were not significant. Additionally, patients with night sweats had a significantly lower risk of developing TD (OR: 0.446, 95% CI: 0.224–0.868, *p*=0.019). Forgetfulness was near significance (OR: 2.356, 95% CI: 0.944–6.205, *p*=0.072). Elevated AST levels significantly increased the risk of TD (OR: 5.437, 95% CI: 1.572–22.42, *p*=0.011). Elevated HCRP levels also significantly increased the risk of TD (OR: 5.544, 95% CI: 2.521–13.145, *p*<0.001). Although the presence of Ro52 antibodies did not reach significance (OR: 2.081, 95% CI: 0.78–5.886, *p*=0.152), it indicated a potential correlation. In summary, pulmonary disease, night sweats, AST, and HCRP are important predictive factors for TD. Particularly, high levels of HCRP and AST significantly increase the risk of TD, while night sweats are associated with a lower risk of TD.

**Table 2:** Predictive factors for thyroid risk in patients with primary Sjögren’s syndrome.

Intercept and variable	Prediction model		p
	β	OR	
(Intercept)	-0.907	0.404	0.231
Pulmonarydisease	0.891	2.437	0.035
Hyperuricemia	-2.088	0.124	0.078
Drynose	-0.634	0.531	0.131
Drythroat	-0.652	0.521	0.055
Anxiety and irritability	-0.276	0.759	0.539
Nightsweats	-0.807	0.446	0.019
Forgetfulness	0.857	2.356	0.072
AST	1.693	5.437	0.011
HCRP	1.713	5.544	<0.001
Ro52	0.733	2.081	0.152

AST, Aspartate Aminotransferase; HCRP, High-sensitivity C-Reactive Protein.

### 3.4 Establishment of the thyroid risk nomogram

**Figure 4:**
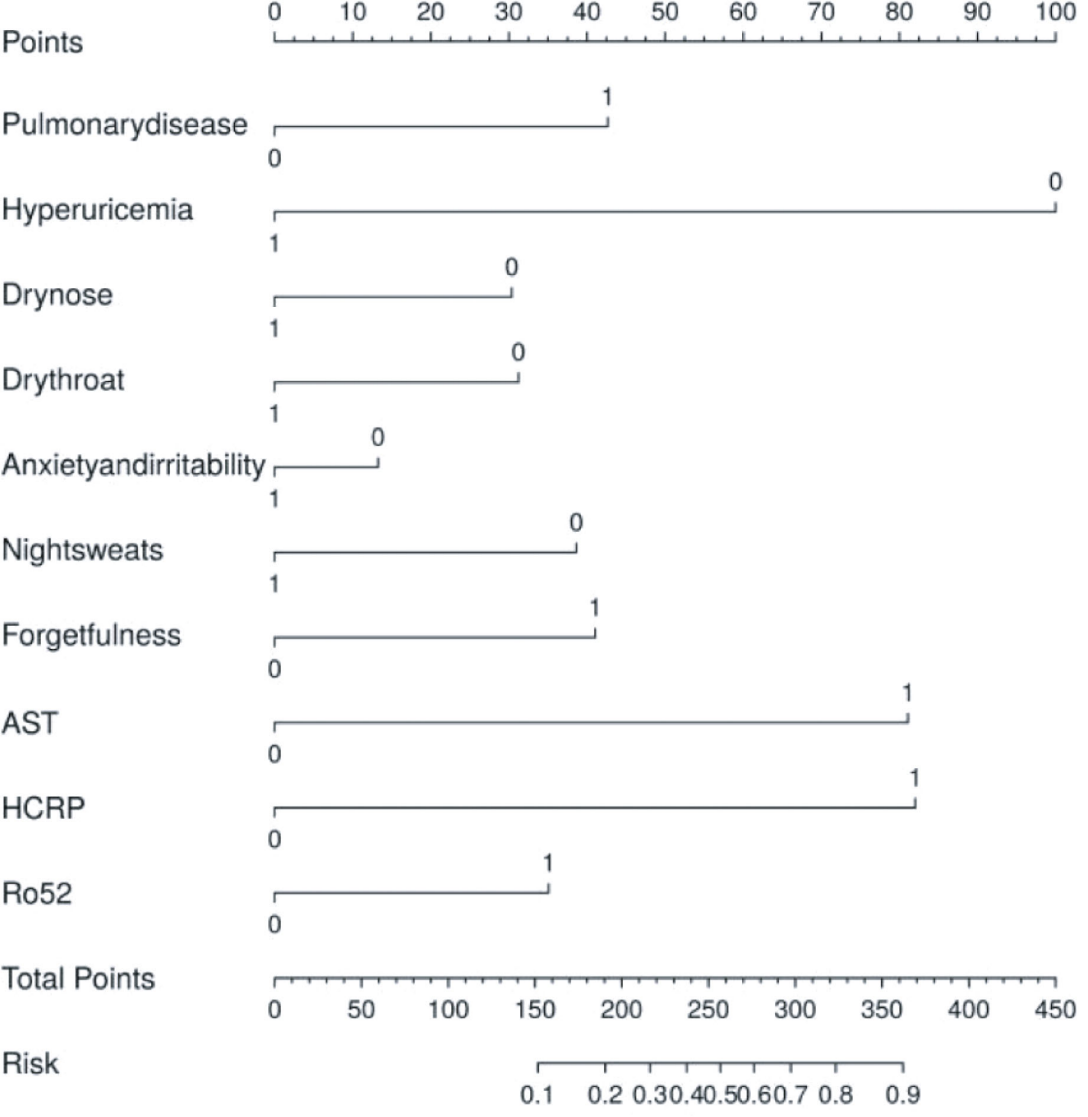
Model establishment and the nomogram. The thyroid risk nomogram was developed within the cohort, incorporating HCRP, pulmonary disease, dry throat, forgetfulness, night sweats, hyperuricemia, dry nose, anxiety and irritability, Ro52, and AST. AST, Aspartate Aminotransferase; HCRP, High-sensitivity C-Reactive Protein.

To facilitate clinical assessment, we developed a nomogram based on 10 significant risk factors, displayed in image form (**Figure 4**). The nomogram model demonstrated satisfactory predictive accuracy with a concordance index of 0.788 (95% CI: 0.726–0.85). The points for each risk factor range from 0 to 100, with a total score ranging from 0 to 450.

### 3.5 Assessment of the thyroid risk nomogram

**Figure 5:**
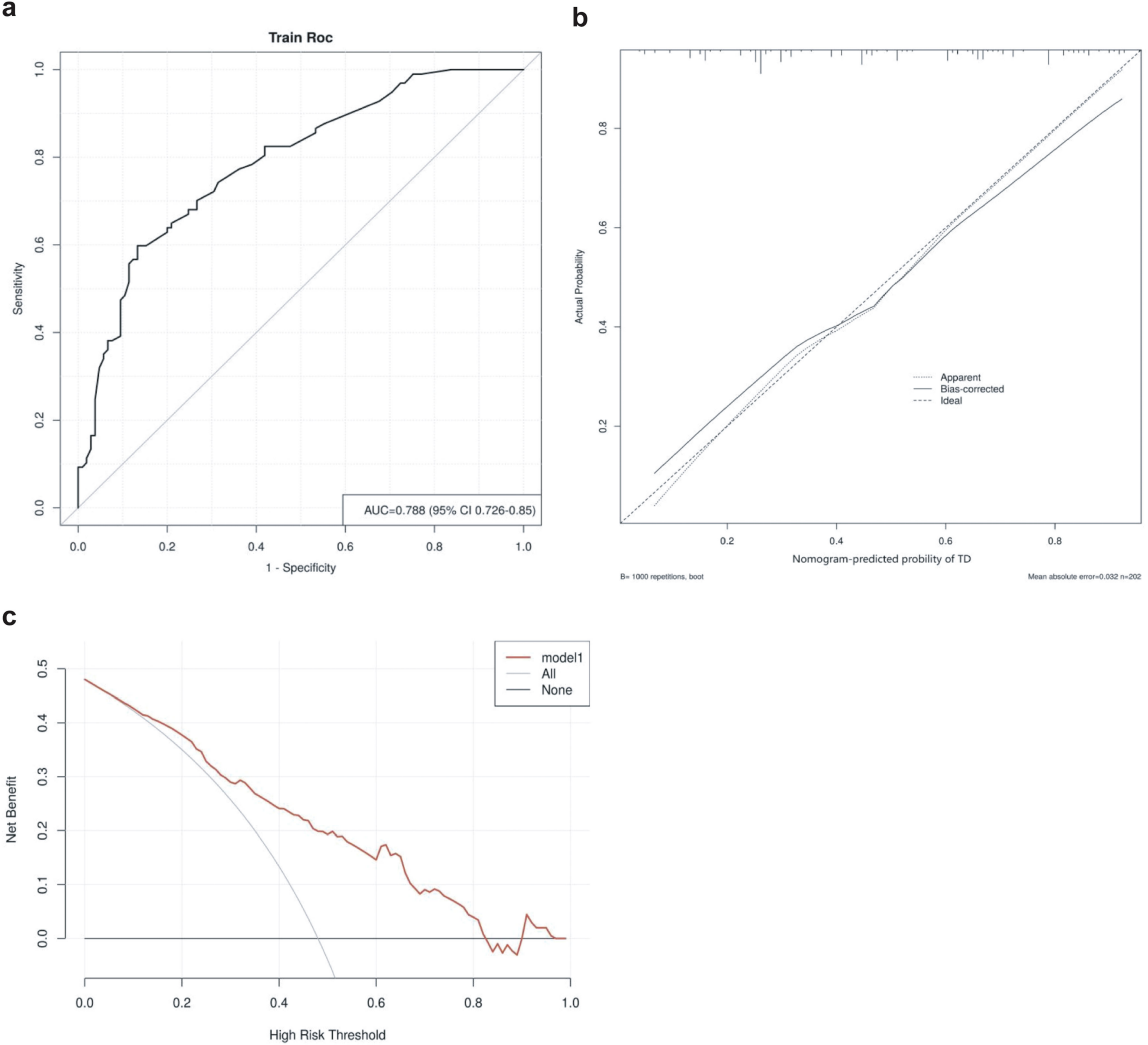
**(A)** Receiver operating characteristics curve for the thyroid risk nomogram in the Cohort. The area under the curve (AUC) is 0.788 (95% confidence interval: 0.726–0.85). **(B)** Calibration curve for the thyroid risk nomogram prediction in the cohort. The x-axis represents the predicted thyroid risk, while the y-axis represents the actual diagnosed thyroid involvement. The diagonal dashed line represents the perfect prediction of an ideal model. The solid line indicates the performance of the nomogram, with a closer proximity to the diagonal dashed line signifying better predictive accuracy. **(C)** Decision curve analysis for the thyroid risk nomogram. The y-axis represents net benefit.

The area under the ROC curve (AUC) for the thyroid risk nomogram is 0.788, indicating a relatively good predictive capability (**Figure 5A**). The calibration curve of the nomogram used to predict thyroid risk in patients with pSS demonstrated good concordance (**Figure 5B**). The Brier Score of the calibration curve was 0.187, indicating that the model has relatively good predictive performance. The decision curve analysis demonstrates that the thyroid risk nomogram provides more benefit than the intervention strategy when the threshold probability for a patient and a clinician is greater than 10% and less than 82%, respectively (**Figure 5C**). The net benefit of the thyroid risk nomogram is comparable to that of several overlapping sections within this range.

## 4 Discussion

In this study, a retrospective analysis was conducted on 202 patients with pSS, leading to the identification of ten key risk factors associated with thyroid involvement: HCRP, pulmonary disease, pharyngeal dryness, forgetfulness, night sweats, hyperuricemia, nasal dryness, anxiety, Ro52 antibodies, and AST. Based on these characteristics, we developed and validated an individualized nomogram to predict the risk of thyroid involvement in patients with pSS. The nomogram demonstrated good discrimination and calibration, with a C-index of 0.788, indicating high predictive accuracy. Decision curve analysis further confirmed the potential clinical utility of the nomogram in guiding intervention strategies. The findings suggest that this predictive tool can assist in early identification of high-risk patients, enabling timely clinical intervention and potentially improving patient outcomes.

In patients with pSS, elevated levels of AST may be associated with an increased risk of thyroid involvement. AST serves as a marker for liver function, with levels typically elevated in the blood in response to liver damage. However, AST is also present in other tissues, including the heart and skeletal muscle, where injury can similarly increase AST levels. In autoimmune conditions such as pSS, chronic inflammation and immune-mediated tissue damage may lead to increased AST levels. Abnormal levels can be observed in Graves’ disease patients, with studies indicating that 29% of 146 Graves’ patients had at least one instance of abnormal AST (25). In Graves’ disease, liver enzyme abnormalities are commonly seen with GGT, followed by ALT, ALP, and then AST. While elevated serum ALP is most frequently observed in hyperthyroid patients, increases in AST and ALT are also common (26). In hypothyroid patients, serum liver enzymes, including AST, often exhibit abnormalities. Hypothyroidism may lead to a slight increase in serum ALT and GGT concentrations, potentially associated with impaired lipid metabolism and hepatic steatosis reported in hypothyroidism (27). Furthermore, increases in AST and LDH may be related to myopathy induced by hypothyroidism (28). However, our study did not identify ALT as a potential risk factor, a discrepancy that might be attributed to the small sample size and variability in disease activity. In patients with pSS, the heightened risk of thyroid events may be associated with systemic inflammation and immune-mediated injury. It could be hypothesized that AST levels in the blood may indirectly reflect this state of systemic inflammation, potentially linking to the risk of thyroid events. However, there is currently no direct evidence implicating AST as a key independent risk factor for thyroid events in patients with pSS. Further research is required to delineate the relationship between AST and thyroid disease in patients with pSS. Future studies should consider large-scale prospective cohort studies to identify potential connections between AST levels and thyroid events in patients with pSS and explore the possible biological mechanisms involved.

In patients with pSS, the positivity of anti-Ro52 antibodies may be associated with an increased risk of thyroid involvement. Ro52, also known as TRIM21, is an E3 ubiquitin ligase that primarily functions in the cytoplasm but can translocate to the nucleus in inflammatory environments and participate in the regulation of type I interferon production (29). Ro52 and anti-Ro52 antibodies are involved in many autoimmune diseases, particularly rheumatic diseases such as pSS and systemic lupus erythematosus (30). The presence of Ro52 antibodies is one of the key indicators for the diagnosis of pSS (31). Anti-Ro52 antibodies may be associated with an increased risk of AITD in patients with pSS. For instance, the expression of HLA class II molecules such as HLA-DR3, HLA-DQ, and HLA-B8 is higher in AITD (32), and they are also susceptibility genes for SS, with HLA-DR3 being particularly associated with the positivity of anti-Ro52 antibodies (33). Although the mechanisms underlying the development of autoimmune diseases including pSS and the formation of thyroid abnormality-related diseases remain elusive, two important characteristics shared by these two diseases are chronic inflammatory states and metabolic dysfunction. The association of anti-Ro52 antibody positivity with thyroid involvement in our model may underscore the potential role of autoimmune responses in thyroid dysfunction in patients with pSS. This finding may suggest that modulation of autoimmune responses could be key in managing thyroid involvement in patients with pSS. Future research should further explore the mechanism by which Ro52 is involved in thyroid involvement in patients with pSS and how this biomarker can be utilized to optimize treatment strategies. For example, studying how the expression of Ro52 in thyroid cells affects the synthesis and secretion of thyroid hormones, and how anti-Ro52 antibodies affect the function of thyroid cells. Additionally, investigating the interaction between anti-Ro52 antibodies and other thyroid autoantibodies (such as anti-TPO and anti-TG antibodies), and how they collectively affect thyroid function, is also an important direction for future research.

HCRP, a biomarker of inflammation, typically increases in response to inflammation or infection. In autoimmune diseases, including pSS, chronic inflammation may result in sustained elevation of HCRP levels. As an inflammatory biomarker, HCRP may reflect the immune-mediated inflammatory processes, and its elevated levels in patients with pSS may correlate with an increased risk of thyroid disease. Research by Manisha Panchal et al. has shown an increased prevalence of elevated levels of HCRP in patients with subclinical hypothyroidism (34), suggesting a potential association between HCRP and the risk of thyroid disease. The inflammatory signs observed in patients with hypothyroidism are thought to be related to the interplay between IL-6, TNF-α, and IL-1, which may be linked to elevated levels of HCRP during hypothyroidism. During this condition, the absence of thyroid hormones leads to a decreased metabolic rate, potentially affecting the clearance rate of HCRP. Hyperthyroidism may be associated with rapid metabolic processes, which could lead to enhanced adrenergic nervous system activity, stimulation of the immune system, and increased peripheral blood flow, all of which may contribute to increased concentrations of HCRP (35). However, despite these associations, it remains unclear whether HCRP is a direct cause of thyroid events in patients with pSS or if it merely serves as a marker of the inflammatory process. Further research is needed to clarify the exact relationship between HCRP and thyroid disease in patients with pSS, including prospective and interventional studies. Such studies would determine whether elevated levels of HCRP predict an increased risk of future thyroid events in pSS and assess whether interventions to reduce levels of HCRP (such as anti-inflammatory therapy) could reduce the risk of thyroid events in the patients. Should HCRP prove to be a reliable predictor of thyroid involvement in pSS, its clinical monitoring could facilitate early detection of patients at risk and inform preemptive therapeutic strategies.

Lung involvement is common in pSS, with a prevalence of interstitial lung disease (ILD) associated with pSS ranging from 3% to 11% (36, 37). Studies have indicated that patients with pSS with lung involvement may exhibit elevated levels of inflammatory markers, such as erythrocyte sedimentation rate (ESR) and CRP, potentially linking to an increased risk of thyroid disease (38). Obstructive disease in patients with dysfunctional thyroid disorders may arise from the direct impact of hormones on pulmonary function (39). Hypothyroidism can lead to a spectrum of respiratory consequences, from mild dyspnea to overt respiratory failure (40), which may alternatively elucidate symptoms of dry throat and nose in patients. Dryness of the throat and nose in patients with pSS primarily results from impaired glands responsible for saliva and nasal mucus production. Thyroid hormones modulate glandular function, and thus, thyroid dysfunction may exacerbate symptoms of dryness indirectly by affecting glandular secretion. Both pSS and thyroid disease encompass autoimmune and inflammatory processes. Systemic inflammation can lead to widespread tissue damage and dysfunction, including effects on thyroid and pulmonary function. Integrated management for patients with pSS with concurrent thyroid involvement and pulmonary abnormalities may be necessary, including thyroid hormone adjustments and autoimmune response control. For symptoms of dry throat and nose, artificial lubricants and immunomodulatory treatments may be required. Future research should further explore the precise mechanisms linking lung involvement, dry throat and nose, and thyroid involvement in patients with pSS, as well as how these symptoms impact quality of life and disease management. Additionally, the efficacy of targeted treatment strategies, such as immunomodulatory therapy and thyroid hormone replacement therapy in patients with pSS, particularly in improving glandular function, alleviating inflammation, and improving prognosis, should be assessed.

Thyroid hormones exert crucial influence on brain metabolism and function, encompassing the regulation of memory and cognitive faculties. Abnormalities in thyroid function, whether hyperthyroidism or hypothyroidism, have the potential to impair memory and cognitive functions (41), leading to forgetfulness. Thyroid hormones and retinoids are now recognized as key signals in modulating neuronal plasticity associated with learning, with variations in the expression of transthyretin protein—a blood carrier for these biomodulatory factors—being characteristically associated with the consolidation of memory traces (42). In patients with pSS, cognitive dysfunction is frequently reported and may be related to systemic inflammation, immune-mediated damage, and other comorbidities (43). Approximately 20% of SS patients exhibit involvement of the central nervous system (CNS). Cognitive and behavioral symptoms of CNS SS include forgetfulness and mental sluggishness, progressing monthly (44, 45). However, a longitudinal study on cognitive decline in SS patients found no significant cognitive deterioration over a 7-year follow-up period (46). Hypothyroidism is not only a potential factor for mood and behavioral changes but also associated with a decline in cognitive function. Its clinical manifestations feature a slowing and inhibition of higher neural activities, sluggish and sustained thinking and speech, and a reduction in memory, sometimes resembling coma, accompanied by symptoms of weakness syndrome and organic dementia (47). Furthermore, hypothyroidism may accelerate the progression of neurodegenerative diseases, increasing the risk of dementia, including Alzheimer’s disease (48, 49). Patients with overt hypothyroidism often exhibit anxiety, inattention, disorientation, impaired learning and perception, and a general decline in intellectual, linguistic, psychomotor, and executive functions (50–52). However, forgetfulness, as a nonspecific symptom, has not yet established a direct link with thyroid events in patients with pSS. Neurological involvement in patients with pSS is diverse, including peripheral, autonomic, and central nervous systems. Future research is needed to investigate the molecular pathways linking thyroid dysfunction with cognitive impairment in pSS and identify potential early interventions to prevent or slow cognitive decline.

Patients with pSS have a significantly higher prevalence of psychiatric disorders compared to the general population (53). Anxiety, the most prevalent subjective symptom, substantially impacts their health, increasing the risk of cardiovascular diseases (53), linking with various psychological disorders (54), impairing work performance (55), and potentially worsening the disease. Reduced salivary secretion raises the risk of dental caries and oral candidiasis in patients with pSS, who also suffer from tooth loss and severe periodontal damage, causing painful chewing (12, 56, 57). This may lead to avoidance of chewing, further deteriorating dental health. Moreover, dry throat and mouth in patients with pSS can impair functions such as chewing, speaking, or swallowing, and cause a sensation of choking, thereby inducing anxiety (58). As the disease progresses, anxiety or depressive moods can intensify dysphagia. Dysphagia not only affects eating but may also cause patients to avoid public eating due to the fear of choking or aspiration pneumonia, reducing quality of life and increasing anxiety. Thyroid hormones play a crucial role in mood regulation, and thyroid dysfunction, particularly hyperthyroidism, often presents with anxiety and irritability. However, no association was found between anti-thyroid antibodies and anxiety or depression in a population-based study. Night sweats, a nonspecific symptom, are associated with mood disorders and hyperthyroidism and may be influenced by physical conditions or anxiety states (59). Lack of understanding of pSS can exacerbate anxiety and depression. Therefore, disease education for patients with pSS is crucial for alleviating these emotional disorders, which may help reduce the occurrence of thyroid events. Education helps patients understand their condition, learn coping strategies, and reduce social and psychological burdens. However, whether anxiety and irritability in patients with pSS directly increase the risk of thyroid events or merely reflect the psychological burden of the disease requires further research for clarification.

While there is currently no direct evidence to suggest that hyperuricemia is an independent risk factor for thyroid events in patients with pSS, several studies have proposed potential links. Uric acid (UA), the end product of purine metabolism, has serum levels that reflect the balance between purine nucleotide catabolism and UA excretion (60). Thyroid hormones can modulate levels of UA, as hyperuricemia is more common in individuals with subclinical thyroid dysfunction than in those with normal thyroid function (61). Giordano et al. (61) reported a significantly higher incidence of hyperuricemia in patients with both hyperthyroidism and hypothyroidism compared to the general population. It has been suggested that hyperthyroidism might precipitate hyperuricemia by enhancing UA synthesis or diminishing renal clearance, potentially due to accelerated purine nucleotide metabolism during the production of UA (62–64). pSS, an autoimmune disease, is characterized by chronic inflammation and immune cell activation. This inflammatory milieu could promote the production of UA, and the thyroid, being a potential target of immune responses, might be influenced by inflammatory mediators. There might be molecular similarities between thyroid and salivary gland cells, allowing the autoimmune response to target not only salivary glands but also the thyroid, resulting in thyroid dysfunction. Hyperuricemia could serve as a biomarker for autoimmune response activation. Further research is warranted to elucidate these mechanisms and to clarify the link between hyperuricemia and thyroid disease in patients with pSS, which could inform clinical management strategies.

This study introduces the first nomogram designed to predict thyroid involvement in patients with pSS. The tool demonstrated good discrimination and calibration in internal validation, signifying its potential accuracy in risk prediction. Additionally, decision curve analysis substantiated the clinical application value of the nomogram, particularly for guiding decisions on thyroid intervention. Despite these promising findings, the study has limitations. Firstly, this study was conducted in a single medical center, and there may be a delay in the diagnosis of thyroid disease in patients with pSS. Future multicenter studies with larger cohorts could enhance and validate the model. Secondly, while we endeavored to include relevant factors, it is still inevitable that some variables influencing thyroid risk were overlooked. Thirdly, patient demographics of our study, predominantly female, reflect the typical gender distribution in pSS and may not be generalizable. Additionally, the exclusion of patients with incomplete data could introduce bias. Lastly, while internal validation has been performed, the robustness of the nomogram has not been verified in external cohorts and should be evaluated in a broader pSS population. At the same time, the applicability of this model in pSS populations in other regions and countries remains to be determined, which requires further validation through prospective studies in larger samples to ascertain its clinical relevance.

In summary, this study identified key factors—HCRP, pulmonary disease, throat dryness, cognitive issues, night sweats, hyperuricemia, nasal dryness, anxiety, Ro52, and AST—that correlate with thyroid involvement in patients with pSS. We developed a personalized nomogram with proven clinical validity for predicting such risks. This tool allows clinicians and patients to make informed decisions on interventions, including lifestyle modifications and medical treatments. Future research should assess the generalizability of the nomogram across diverse populations and refine it with additional risk factors for enhanced predictive accuracy. Additionally, external validation in other pSS cohorts is necessary to confirm its potential to mitigate thyroid risks and influence clinical management strategies.

## Data Availability

The raw data supporting the conclusions of this article will be made available by the authors, without undue reservation.
